# Analysis of Austrian COVID-19 deaths by age and sex

**DOI:** 10.1007/s00508-020-01707-9

**Published:** 2020-07-03

**Authors:** Martin Posch, Peter Bauer, Alexander Posch, Franz König

**Affiliations:** Section for Medical Statistics, Center for Medical Statistics, Informatics and Intelligent Systems, Medical University of Vienna, Spitalgasse 23, 1090 Vienna, Austria

**Keywords:** COVID-19, Epidemics, Death, Austria, Sex distribution, Age distribution

## Abstract

We analyze the age and sex distribution of the reported COVID-19 deaths in Austria. In accordance with international studies, the Austrian data also suggests that the risk of death increases substantially with age. The observed age dependency of the proportions of registered COVID-19 deaths in relation to the population sizes in the age groups is approximately exponential, similar to the age dependency of the general age specific mortality rate. Furthermore, we compare the general age specific mortality rate in Austria with the estimates of the SARS-CoV‑2 infection fatality rate by Ferguson et al. (2020). The parallels to the general age specific mortality rates do not imply that COVID-19 does not pose an additional risk. On the contrary, it follows from the structure and magnitude of the infection fatality rate that it is substantial, especially for higher age groups. However, since in many cases persons with severe pre-existing conditions are affected, it is not yet possible to estimate what effects COVID-19 will have on life expectancy.

To the best of our knowledge, the most recent published evaluation of Austrian COVID-19 death data by age and sex is based on the 112 registered COVID-19 deaths that were available in the epidemiological reporting system of the Federal Ministry of Social Affairs, Health, Care and Consumer Protection (in the following abbreviated as Ministry of Health) by 15:00 on 31 March 2020 (of the total 128 deaths by that time) [1]. In addition, the current marginal age distribution and marginal gender distribution (but not the gender distribution per age group) of deaths is continuously published on the dashboard of the Ministry of Health [2]. For this analysis we used both the data set from March 31, as well as data from the dashboard including 439 deaths reported by 11:00 on 21 April 2020. In both data sets, the majority of the deceased are men (61% men by 31 March and 58% by 21 April). Most of the deceased are over 60 and 55 years of age, respectively (Fig. 1a and b). When comparing the graphs, the different age categories and total number of deaths should be taken into account. However, the absolute figures must be related to the size of the population in each age group. Then they can be interpreted as current COVID-19 mortality per 10,000 people in each age group in Austria (Fig. 1c and d) accounting for all cases reported to the epidemiological reporting system by 31 March and 21 April, respectively. This illustration shows a clear age trend and the gender difference is even more pronounced (since women are in the majority in the upper age groups of the population). It should be noted that the graphs do not show the infection fatality rate, defined as the proportion of fatalities among infected persons, but the proportion of registered COVID-19 deaths in relation to the general population.

**Fig. 1:**
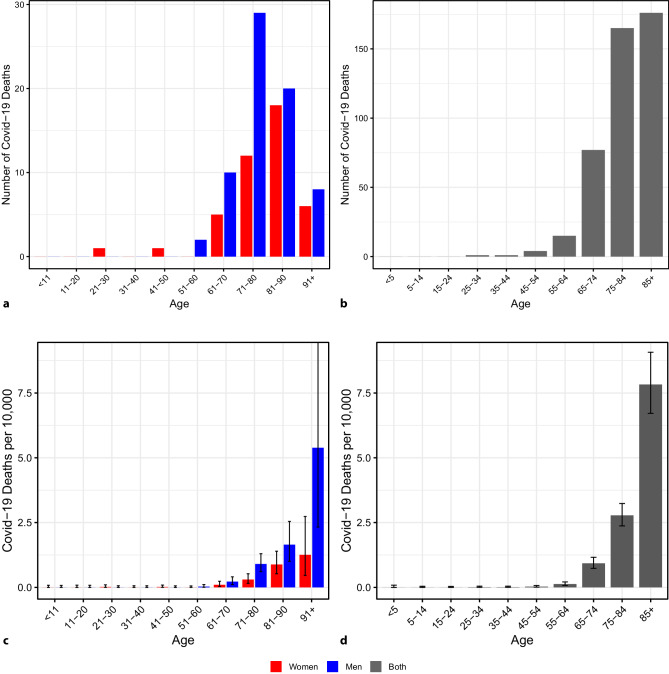
**a** Age distribution and sex of COVID-19 deaths in Austria that were registered up to 31 March 2020. **b** Age distribution of COVID-19 deaths in Austria that were registered up to 21 April 2020 (both sexes pooled). Due to the different age categories and different total number of deaths, the absolute figures are not directly comparable with the left figure. **c** COVID-19 deaths per 10,000 in the respective age and sex group in the Austrian population (deaths reported up to 31 March 2020) and 95% confidence intervals. 2018 Population size data from [9]. **d** As **c** but with other age categories, sex groups pooled, and including deaths reported until 21 April

The age dependency of mortality rates is not unusual. Also, the normal mortality risk depends strongly on age. The mortality tables show for each age and sex the number of people who die in the next year of life (Fig. 2a, red and blue lines) per 10,000 people in that age and sex group. The risk of mortality is high at birth, but then drops below 0.01% by age 7. After that, it increases again, especially among the more risk-taking young men. The risk of men remains higher than that of women until old age and only approaches that of women at the end of the curve. If we compare this normal mortality risk with the COVID-19 mortality until 31 March or 21 April, respectively (Fig. 2a and b), we see a similar increase (although the number of COVID-19 deaths registered by the respective survey date scaled to the population size is orders of magnitude lower than the annual mortality rates). The proportion of Austrians who die from COVID-19 by a given date depends on how many people are infected and the risk of death among those infected, the so-called infection fatality rate.

**Fig. 2:**
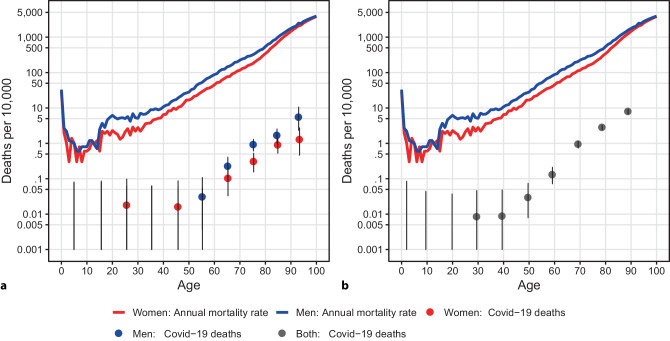
**a** *Red* (women) and *blue* (men) *lines*: number of people per 10,000 persons in the Austrian population who normally die within the next year of life (average of the years 2015–2017 from [9]). *Red* (women) and *blue* (men) *dots*: number of persons per 10,000 in the Austrian population who died with COVID-19 and were registered in the epidemiological reporting system by 31 March 2020. These are the numbers from Fig. 1c, plotted on a log scale. Zero counts are not plotted. The numbers are plotted at the mean ages of the Austrian population in the respective age categories. Due to the exponential increase of the risk with age, this gives a slightly positively biased estimate of the number of deaths at the mean age. The vertical lines represent 95% confidence intervals. Lower bounds are cut off at 0.001. **b** *Red* and *blue lines* as in **a**. *Grey dots*: as the *blue* and *red dots* in **a** but for deaths reported until 21 April and pooled sexes

However, estimating infection fatality rates is complex [4, 5]. A simple, but not very reliable estimator is the proportion of observed deaths among those who tested positive. However, the positively tested are only a part of all infected persons and it is difficult to quantify the dark figure of undetected infections that are mild or even without symptoms. It is difficult to determine the number of undetected cases of infected persons, as it depends heavily on the number of tests and the testing strategy. Only representative antibody tests will provide reliable estimates. Therefore, instead of infection fatality rates, case fatality rates (the number of deaths per registered cases) are often considered instead. But these are also difficult to estimate during an outbreak of an epidemic because many of those who test positive are still ill when the data is collected and may die within the next few days.

The case fatality rates however, depend sensitively on the number of undetected cases, which explains part of the differences between the numbers reported in different countries. An indication for the infection fatality rate is provided by the cases on the Diamond Princess, the cruise ship on which all passengers were tested and a mortality rate of 1.5% (11 out of 712 tested positive) was observed. However, the risk of death is strongly age dependent. Scientists at Imperial College London [3] used a model-based approach to estimate the age-dependent risk for infected persons to die from COVID-19. Sir David Spiegelhalter, professor of risk communication at the University of Cambridge, observed that these estimated infection fatality rates were roughly similar to the normal annual mortality risk in the United Kingdom [6, 7]. Comparing the infection fatality rate estimates reported in [3] with the normal annual mortality rates for Austria, we see parallels as well – the estimated risk of death with COVID-19 is for the majority of age groups roughly as high as the normal risk of dying within 1–2 years (Fig. 3, black dots). However, the risk is not spread over the entire period, but concentrated on a much shorter interval. We also observe that the number of those who have died in Austria with COVID-19 per population size in the respective age and sex group have a similar exponential age dependency as the estimated infection fatality rates (Fig. 2a, b and 3). On closer inspection, one sees that the increase of the former is somewhat steeper. This could still be consistent with the infection fatality rate estimates if infection rates are higher in the upper age groups. In fact, if the number of people who tested positive are put in relation to the population size, the current data of the Ministry of Health show that the group of people over 85 years of age is overrepresented.

**Fig. 3:**
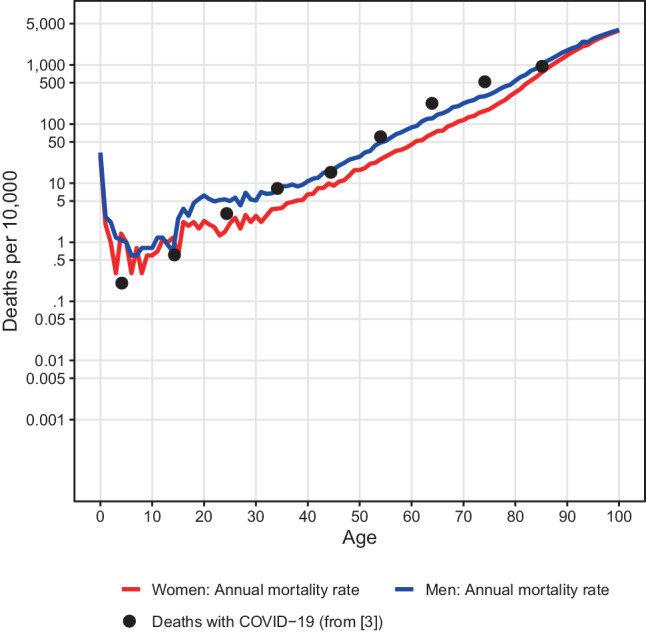
*Red* and *blue lines* as in Fig. 2a and b. *Black dots*: estimated infection fatality rates per age group according to [3] plotted at the mean ages of the age groups for which the estimates were reported. Due to the exponential increase of the risk with age, this gives a slightly positively biased estimate of the number of deaths at the mean age

A misinterpretation would be to conclude from these considerations that the risk of death is not increased. On the contrary, it follows from the structure and magnitude of infection fatality rate that it is substantial, especially for higher age groups. Although it is unclear how many people would have died in the course of the year even without COVID-19 disease, it can be assumed that the risk of dying from COVID-19 is to a substantial extent an additional risk. It is not yet known, however, how many years of life (on average) one loses through a SARS-CoV‑2 infection. Since many seriously ill patients with already shortened life expectancy are affected, the additional effect on the general life expectancy could be less than it would be derived from mortality alone. Although in the long run the loss of years of life may be a more relevant figure than mortality itself, in the short run, especially to assess the social impact, mortality is of greater importance.

Such risk comparisons put the danger of COVID-19 disease into perspective, but it is doubtful whether this pandemic can be perceived as less drastic. These mortality estimates do not take into account the possible overloading of the healthcare system and the associated indirect effects on mortality of other diseases, such as heart attacks, strokes, or cancer, which can no longer be treated appropriately. According to estimates by Imperial College London [3], 5% of infected 40- to 50-year-old people need hospital treatment, a figure that rises to over 25% for those over 80. Of those treated in hospital, about 6% of 40- to 50-year-old people need intensive care and over 70% of those over 80. In order to comprehensively evaluate the effects of the disease, it is also necessary to consider in which state of health, for example, older patients who did not die leave the hospital after intensive care treatment. The long-term consequences of the disease are also not yet known. Therefore, comprehensive measures to control the spread of the epidemic are essential to prevent the collapse of the health system. It is difficult to include the social and economic consequences of the measures, which can also have medical consequences.

Even if deaths are easier to collect than the number of people infected, these figures are not without controversy. Firstly, it is often difficult to determine whether a patient dies from COVID-19 (i.e., the virus is the cause of death) or dies with the virus (i.e., is infected but the cause of death is different). Two figures are currently reported by the Ministry of Health: those of the deceased who tested positive (regardless of the actual cause of death) and those who died from COVID-19. On the other hand, there may also be an unrecorded number, since COVID-19 deaths may not always be recognized as such. More reliable than the number of deaths due to COVID-19 is the total number of deaths. Reports from several countries with stronger outbreaks of the epidemic indicate that the death figures in recent weeks are significantly higher than the long-term averages. But also in Austria, increased death rates, especially among the elderly, have been reported recently [8]. The extent to which the deaths affect people who would have died this year even without COVID-19 may become apparent in the annual statistics.
